# Infantile Fibrosarcoma of Tongue: A Rare Tumor

**DOI:** 10.21699/ajcr.v7i3.432

**Published:** 2016-06-15

**Authors:** Fazal I Wahid, Bakht Zada, Gul Rafique

**Affiliations:** Department of ENT, Head and Neck Surgery Medical Teaching Institute (MTI), Lady Reading Hospital (LRH), Peshawar-Pakistan

**Keywords:** Fibrosarcoma, Infantile fibrosarcoma, Soft tissue tumor

## Abstract

Infantile fibrosarcoma is very rare tumor and seldom occurs in oral cavity. Overall good prognosis is reported in more than 80% cases. We present a 5-month old female patient with swelling of the tongue for four months. This was excised completely. Histopathology and immunohistochemistry revealed it as infantile fibrosarcoma of grade II.

## CASE REPORT

A 5-month old female infant presented with progressive swelling of the tongue since one month of age. There was no associated difficulty in swallowing or breathing. On examination, there was a firm, diffuse swelling about 3cm x 4cm in dimension, involving left side of the tongue and extending to midline. Mucosa over the mass was intact and margins were ill-defined (Fig. 1). There was no associated cervical lymphadenopathy. Ultrasonography revealed heterogeneous mass in the tongue sparing the floor of mouth. Fine needle aspiration was inconclusive. At operation adrenaline mixed local anesthetic was injected at the incision site to minimize the bleeding. Linear incision was made on the left lateral border of the tongue. The mass was excised and wound stitched with polyglycolic 3/0. Patient was put on injectable antibiotics for 48 hours with mouth cleansing antiseptic oral spray. Specimen was sent for histopathology which was reported as infantile fibrosarcoma of grade II. The margins of the excision were tumor free. Immunohistochemical staining showed positive staining for SMA and CD34 which also supported the diagnosis. Patient was reviewed postoperatively and referred to oncologist for chemo-therapy (Fig. 2). At three months follow up patient was disease free after receiving chemotherapy.

**Figure:**
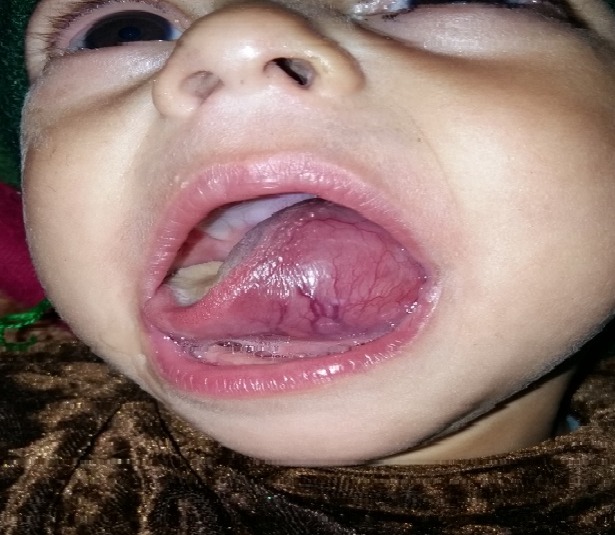
Figure 1:Five month old baby with swelling of the tongue.

**Figure:**
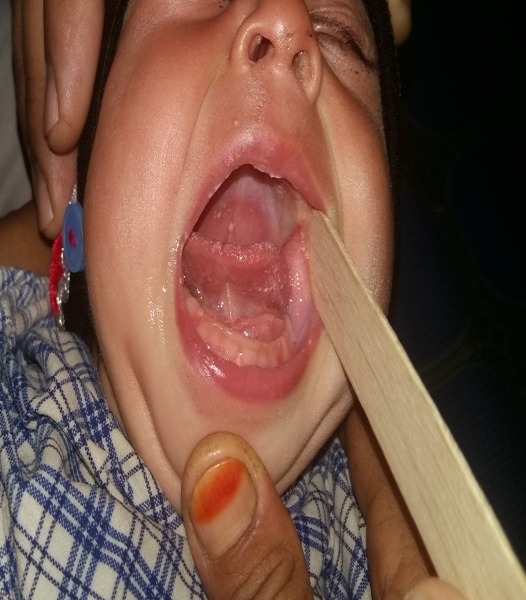
Figure 2:Post-operative picture after excision of the mass.

## DISCUSSION

Fibrosarcomas are malignant tumors predominantly originating from soft tissues.[1] Fibrosarcomas are commonly found in the extremities, trunk, and head and neck regions in descending order of frequency. Among all the fibrosarcomas, only 0.05 % occur in head and neck region, of which 23% occur in oral cavity.[2] Rarely it arises from the tongue.[2-5] There are two types of fibrosarcomas, infantile or congenital (IF) and adult form. The presentation of congenital fibrosarcoma is usually in early age as in our case. Clinical features depend upon the size, site and grade of the tumor. However, it presents as slow growing painless mass.[6] Feeding and breathing difficulties are the main concerns in case of intraoral mass lesions. In our patient the mass was also increasing slowly in size but it was not causing any feeding or breathing difficulties. The exact cause of IF remains obscure, however genetic malformation and radiation may contribute.[3]

CT scan or MRI may point a solid mass but the exact diagnosis can only be made on histopathology powered by immunohistochemistry as we diagnosed in our case.[2,6,7] Infantile fibrosarcoma can be graded into low, intermediate and high grade depending on severity of cellularity, nuclear pleomorphism and mitosis.[3] Infantile fibrosarcoma must be kept in the differentia diagnosis of other soft tissues tumors like rhabdomyosarcoma and Infantile myofibromatosis and its variants.[4,8] Our case was of grade II on histopathology. The management of IF is a multidisciplinary which includes surgery, chemotherapy and radiotherapy with rehabilitation if indicated.[4] Surgery is the mainstay of treatment for IF. In our case the mass was excised apparently completely and then patient was referred to oncologist. The success of treatment of IF depends on age of the patient, extent and site, grade and stage of the disease, response to the treatment and associated side effects of the medications. Overall good prognosis of IF is more than 80%, if treated in time.[8] Our patient is doing well till date. In conclusion, although infantile fibrosarcoma is a rare tumour, it must be kept in differential diagnosis of soft tissue tumors presenting in infancy. Surgery is the mainstay of treatment for infantile fibrosarcoma that can be complemented with chemo-radiotherapy depending upon its stage.

## Footnotes

**Source of Support:** Nil

**Conflict of Interest:** None declared

